# Immunomodulatory Effects of *Symplectoteuthis oualaniensis* Protamine and Its PEG Derivative on Macrophages: Involvement of PI3K/Akt Signaling, Redox Regulation, and Cell Cycle Modulation

**DOI:** 10.3390/antiox14040437

**Published:** 2025-04-04

**Authors:** Na Li, Yida Pang, Jiren Xu, Jeevithan Elango, Wenhui Wu

**Affiliations:** 1 Department of Marine Pharmacology, College of Food Science and Technology, Shanghai Ocean University, Shanghai 201306, China; d220300085@st.shou.edu.cn (N.L.); 2334219@st.shou.edu.cn (Y.P.); m240451270@st.shou.edu.cn (J.X.); 2 Department of Biomaterials Engineering, Faculty of Health Sciences, UCAM-Universidad Católica San Antonio de Murcia, Guadalupe, 30107 Murcia, Spain; 3 Center of Molecular Medicine and Diagnostics (COMManD), Department of Biochemistry, Saveetha Dental College and Hospitals, Saveetha Institute of Medical and Technical Sciences, Saveetha University, Chennai 600077, India; 4 Marine Biomedical Science and Technology Innovation Platform of Lin-gang Special Area, Shanghai 201306, China; 5 Putuo Branch of International Combined Research Center for Marine Biological Sciences, Zhoushan 316104, China

**Keywords:** protamine, PI3K-Akt signaling pathway, immune modulation, reactive oxygen species (ROS)

## Abstract

Protamine is a promising marine-derived bioactive compound that is highly arginine-rich and has demonstrated unique advantages in medical and biological research. This study, for the first time, investigates the molecular mechanisms underlying the immunomodulatory effects of Salmon Protamine Sulfate (SPS), *Symplectoteuthis oualaniensis* Protamine (SOP), and its polyethylene glycol (PEG) derivative (SOP-PEG) on RAW264.7 macrophages. The results demonstrate that both SOP and SOP-PEG significantly enhance the proliferation of RAW264.7 cells by promoting the secretion of pro-inflammatory cytokines and nitric oxide (NO), increasing ROS production, and improving antioxidant capacity, in comparison to SPS. Elevated ROS levels play a crucial role in enhancing macrophage immune activity, while the enhanced antioxidant defense mechanisms help maintain redox homeostasis and protect against oxidative stress-induced cellular damage. A Western blot analysis reveals that SOP and SOP-PEG notably regulate the expression of key proteins associated with the PI3K/Akt signaling pathway and anti-apoptotic mechanisms. Furthermore, a flow cytometry analysis indicates a significant increase in the G2/M-phase cell population in the treatment groups, which is corroborated by Western blot data showing alterations in critical regulatory proteins. Notably, SOP-PEG exhibits the strongest effects in regulating macrophage immune activity, which can be attributed to the enhanced stability and prolonged bioactivity resulting from the PEGylation of SOP. This comprehensive study reveals how SOP and SOP-PEG enhance macrophage immune function through multiple mechanisms, including PI3K/Akt activation, redox regulation, and cell cycle modulation. It provides valuable insights and a theoretical foundation for their potential applications in immunotherapy and immune regulation.

## 1. Introduction

In recent years, the role of the immune system in infection defense, tumor surveillance, and tissue repair has garnered significant attention [1,2,3]. Macrophages, as key effector cells of the innate immune system, play a direct role in the phagocytosis and elimination of pathogens [4,5,6]. In addition, they regulate the activity of other immune cells by secreting cytokines, making them indispensable in immune responses [7,8]. Investigating the regulatory mechanisms of macrophage function is crucial for enhancing immune defense capabilities and developing novel immunotherapies.

Proteins, owing to their unique biological activities, have demonstrated significant potential in enhancing host immunity and modulating immune system functions [9,10]. Marine organisms, particularly deep-sea invertebrates, are increasingly recognized as important sources of bioactive proteins for the development of novel immunomodulators [11,12]. Research has demonstrated that marine-derived proteins and peptides play critical roles in immune system regulation [13,14]. For instance, Dai et al. reported that squid collagen (STC-II) alleviated degenerative osteoarthritis by suppressing the STAT1 signaling pathway in macrophages [15]. This mechanism highlights the potential of STC-II in modulating macrophage-mediated inflammation and its therapeutic implications for inflammatory joint disorders. In addition, polysaccharides derived from the coral *Pseudopterogorgia americana* [16] and bioactive molecules such as proteins and peptides from marine sources, including *Penaeus monodon* [17], *Mytilus galloprovincialis* [18], and *Mytilus coruscus* [19], have demonstrated remarkable immune-regulatory properties across a range of clinical conditions.

*Symplectoteuthis oualaniensis* (*S. oualaniensis*), a deep-sea organism adapted to extreme environments such as high pressure and low oxygen, exhibits remarkable growth and reproductive advantages, making it a promising protein resource [20]. Among its bioactive compounds, protamine, a unique class of basic proteins, has attracted significant research interest due to its distinctive structure and functions. They demonstrate exceptional structural stability and resilience, alongside notable bioactivities in immunomodulation, anti-inflammatory, and antioxidant processes [21,22,23]. These characteristics position protamine as an ideal candidate for studying macrophage function regulation [24,25]. Furthermore, the high safety profile and broad biological effects of protamine provide favorable conditions for its potential applications in the biomedical field [26]. Ramzan et al. found that protamine sulfate induced mitochondrial hyperpolarization and a subsequent increase in reactive oxygen species (ROS) production [27]. However, the immunomodulatory effects of *S. oualaniensis* protamine (SOP) have been rarely studied.

In addition, the polyethylene glycol (PEG) conjugation of proteins is widely recognized as an effective strategy to enhance the stability and safety of therapeutic agents, making it a preferred approach for developing protein-based therapies [28,29,30]. Accordingly, we proposed the PEGylation of protamine. Our previous studies demonstrated that PEGylation significantly altered the surface morphology, secondary structure composition, and thermal decomposition behavior of protamine, while notably improving its thermal stability [31]. By PEGylating protamine, its pharmacological properties can be further optimized, expanding its potential applications [32,33]. However, current research on the immunomodulatory effects of protamine and its PEGylated derivatives in macrophages remains limited. Furthermore, reactive oxygen species (ROS), as critical signaling molecules, play a pivotal role in regulating macrophage activity and inflammatory responses [34]. Simultaneously, the PI3K-Akt signaling pathway serves as a key molecular mechanism mediating cell survival, proliferation, and functional modulation [35,36]. These findings provide a theoretical basis for further investigating the mechanisms underlying the effects of protamine and its PEGylated derivatives.

This study aims to investigate the immunomodulatory effects of the arginine-rich basic protein *S. oualaniensis* protamine (SOP) and its PEGylated derivative (SOP-PEG) on macrophages, as well as elucidate their underlying molecular mechanisms. These results are anticipated to offer fresh theoretical perspectives on the potential applications of protamine in immunotherapy and pave the way for advancing protein-based immunomodulators.

## 2. Materials and Methods

### 2.1. Materials and Reagents

SOP and SOP-PEG were prepared in our lab, with detailed extraction, purification, and PEGylation methods in published studies [31]. SOP (14.3 kDa) was obtained from spermary tissue via HCl extraction, ethanol precipitation, dialysis, lyophilization, and Sephadex G-50/CM Sepharose purification. SOP-PEG (18.5 kDa) was synthesized by reacting SOP with PEG2000 at room temperature for 24 h, followed by dialysis and lyophilization. Salmon protamine sulfate (SPS) was purchased from Sigma-Aldrich (Catalog No. 53597-25-4, St. Louis, MO, USA). RAW264.7 cells were obtained from the Type Culture Collection of the Chinese Academy of Sciences (Shanghai, China). The following reagents were used in the study: 0.25% trypsin-EDTA (1×), penicillin/streptomycin (P/S), FBS, and PBS, all purchased from Thermo Fisher Scientific (Gibco, Waltham, MA, USA). Neutral red staining solution for live cells was obtained from Beyotime (Shanghai, China). RNA and qPCR-related reagents were purchased from Accurate Biotechnology Co., Ltd. (Hunan, China). For protein electrophoresis and Western blotting, Protein Standard Marker, Sample Buffer, and Tris/Glycine/SDS were purchased from Bio-Rad Laboratories Inc. (Hercules, CA, USA). PVDF membranes was obtained from Millipore (Billerica, MA, USA). The primary antibodies used included PI3K, Akt, p-Akt, Bcl-2, Bax, P53, CDK1, PLK1, and cyclin B1, all diluted at a ratio of 1:1000. Unless stated otherwise, all additional reagents were obtained from Sigma-Aldrich (St. Louis, MO, USA).

### 2.2. Culture of RAW 264.7 Macrophages

RAW264.7 cells were maintained in DMEM containing 10% FBS and 1% penicillin/streptomycin under humidified conditions at 37 °C with 5% CO_2_ and 95% air. To support optimal cell proliferation, the culture medium was refreshed every two days.

### 2.3. Cell Viability Assay

Cell viability was assessed using the CCK-8 (AbMole BioScience, Harvard, TX, USA) following the manufacturer’s instructions. RAW264.7 cells (1 × 10^4^ per well) were seeded in 96-well plates and incubated at 37 °C with 5% CO_2_ for 24 h. Cells were then treated with different concentrations of protamine (5–60 μM; SOP, SOP-PEG, and SPS) for another 24 h, while control cells received only culture medium. After treatment, the medium was removed, and cells were washed with PBS. Then, 100 μL of medium containing 10% CCK-8 reagent was added, followed by incubation at 37 °C in the dark for 2 h. Optical density (OD) values were measured to determine cell viability, with untreated controls set as 100%.

### 2.4. Enzyme-Linked Immunosorbent Assay (ELISA)

RAW264.7 cells were seeded at a density of 1 × 10^4^ cells/well in a 96-well plate and incubated at 37 °C with 5% CO_2_ for 24 h. After the cells adhered to the plate, the medium was replaced with medium containing different concentrations of SOP, SOP-PEG, and SPS (10, 20, 40 μM) and further incubated for 24 h. The culture supernatant was then collected, centrifuged at 12,000 rpm for 10 min at 4 °C, and the supernatant was used for analysis. Inflammatory cytokine (IL-1β, IL-6, TNF-α) levels were measured using an ELISA kit (MEIMIAN, Yancheng, China). According to the kit instructions, standard and sample wells were set up, with 50 μL of standard or 10 μL sample + 40 μL diluent added to the wells, and the blank wells were left empty. Except for the blank wells, 100 μL of HRP-labeled antibody was added to each well, sealed with an adhesive membrane, and incubated at 37 °C for 60 min. After discarding the liquid, the wells were washed five times. Subsequently, 50 μL of substrate A and B was added to each well, and the plate was incubated at 37 °C in the dark for 15 min. After adding 50 μL of stop solution, the absorbance at 450 nm was measured, and the cytokine concentrations were calculated based on the standard curve. NO levels were measured using the Griess reagent method (MEIMIAN, Yancheng, China). Fifty microliters of supernatant was added to a 96-well plate, and Griess Reagent I and II were sequentially added according to the kit instructions. The plate was mixed and incubated in the dark at 37 °C for 10 min. The absorbance at 540 nm was measured, and the NO concentration was calculated using the standard curve. The experimental results were normalized to protein content.

### 2.5. Phagocytosis Assay

RAW264.7 cells were seeded into 96-well plates (1 × 10^5^ cells/mL) and incubated for 24 h. Cells were then treated with protamine (SOP, SOP-PEG, SPS) or a control medium for another 24 h. Following treatment, the medium was discarded, and 100 μL of 0.1% neutral red solution in PBS was added. After 1 h of incubation, the cells were rinsed with PBS and then stained overnight with 100 μL of 1% acetic acid in 50% ethanol. The absorbance at 540 nm was recorded using a microplate reader (BioTek, Winooski, VT, USA).

### 2.6. Reactive Oxygen Species (ROS) Measurement

The release of ROS by macrophages was analyzed using a ROS assay kit (Beyotime, Shanghai, China). RAW264.7 cells were plated in 6-well plates at 1 × 10^5^ cells per well and exposed to the specified concentrations of SOP, SOP-PEG, SPS, or NAC (5 mM), either alone or in combination, for 24 h. PBS was used as the vehicle control. Following treatment, the cells were harvested, centrifuged, and incubated with 10 μM DCFH-DA at 37 °C for 30 min. Subsequently, the cells were washed twice with PBS to remove the excess probe. The average fluorescence intensity was measured using a FACSCelesta flow cytometer (BD Biosciences, San Jose, CA, USA), and the results were analyzed with FlowJo software (version 10.8.1).

### 2.7. Assessment of Total Antioxidant Capacity and Superoxide Dismutase Activity

RAW264.7 cells (2 × 10^5^ cells/well) were treated with SPS, SOP, and SOP-PEG for 24 h, then lysed in PBS, centrifuged, and the supernatant collected for analysis.

Total Antioxidant Capacity (T-AOC) [37]: T-AOC was assessed using the ABTS method with a Total Antioxidant Capacity Assay Kit (Beyotime, Shanghai, China). After preparing and diluting the ABTS stock solution, 200 μL was added to the supernatant, incubated for 5 min, and OD at 734 nm was measured.

Superoxide Dismutase (SOD) [38]: SOD activity was measured using the WST-8 method with a Total Superoxide Dismutase Assay Kit (Beyotime). After adding the WST-8/enzyme solution, incubation for 30 min, and mixing, OD was measured at 450 nm.

### 2.8. Quantitative Real-Time Polymerase Chain Reaction (qRT-PCR)

The qRT-PCR analysis was performed as described in our previous reports [39]. Total RNA was extracted using the SteadyPure Quick RNA Extraction Kit (Accurate, Changsha, China) and its quantity and purity assessed via a Nanodrop spectrophotometer (ThermoFisher, Waltham, MA, USA). cDNA synthesis was performed with 1 µg of RNA using the Evo M-MLV RT Mix Kit (Accurate, Changsha, China). Quantitative RT-PCR was carried out in a 20 µL system with the SYBR Green Premix Pro Taq HS qPCR Kit, following standard cycling conditions. GAPDH was used as the internal control, and target gene expression was calculated using the comparative CT method. The specific primers were designed and synthesized by Sangon Biotech, with their sequences listed in Table 1.

### 2.9. Western Blot Analysis

The Western blot analysis was performed as described in our previous reports [40]. Macrophages treated with various protamine concentrations were lysed on ice for 20 min in a RIPA buffer with a protease inhibitor cocktail. After centrifugation, protein concentration was determined using the BCA Protein Assay Kit (TIANGEN, Shanghai, China). Equal protein amounts were boiled in a loading buffer, separated by SDS-PAGE, and transferred to PVDF membranes. Membranes were blocked with 5% milk and incubated with primary antibodies overnight at 4 °C. After washing, HRP-conjugated secondary antibodies were applied, and chemiluminescence was detected. Protein bands were quantified using ImageJ (version 1.54p).

### 2.10. Cell Cycle Assay

RAW264.7 cells (2 × 10^5^ cells/well) were seeded in 6-well plates and treated with SPS, SOP, or SOP-PEG for 48 h. After treatment, the cells were stained with Annexin V-FITC and PI, and a cell cycle analysis was performed using flow cytometry (BD Biosciences, San Jose, CA, USA). Data were analyzed with FlowJo software to quantify cell cycle phases.

### 2.11. Statistical Analysis

Data are presented as mean ± SD of three replicates. Statistical significance was assessed using one-way ANOVA and Duncan’s test (SPSS 17.0), with *p* < 0.05 considered significant.

## 3. Results

### 3.1. Cell Viability Assessment

**Figure 1:**
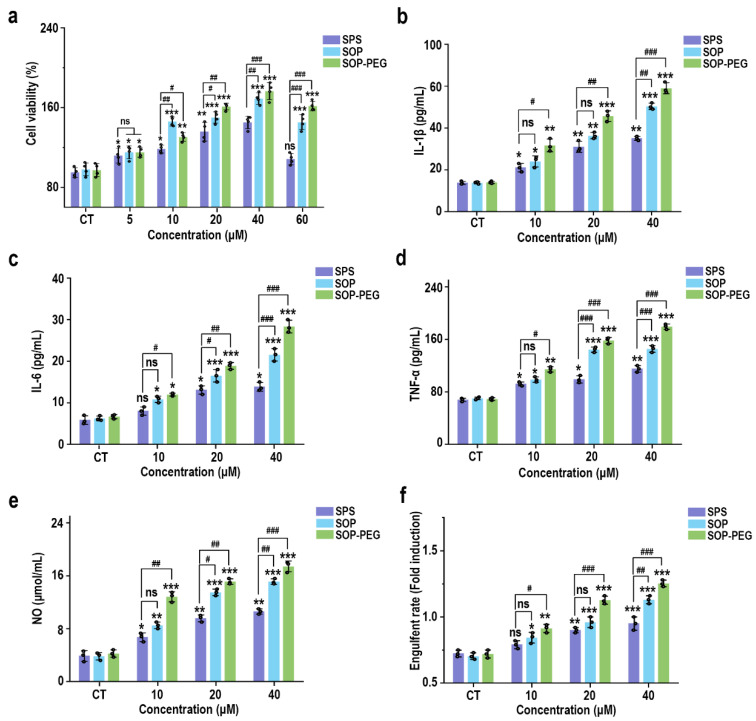
Effects of SPS, SOP, and SOP-PEG on the cell viability (**a**), cytokine secretion levels (IL-1β (**b**), IL-6 (**c**), and TNF-α (**d**)), NO production (**e**), and phagocytosis activity (**f**) of RAW264.7 cells. *, **, and *** represent *p* < 0.05, *p* < 0.01, and *p* < 0.001 compared to the control group, respectively; ^#^, ^##^, and ^###^ represent *p* < 0.05, *p* < 0.01, and *p* < 0.001 for significant differences between two groups. ns indicates no significant difference.

To evaluate the effect of different sources of spermidine (SPS, SOP, and SOP-PEG) on macrophage activity, RAW264.7 cells were incubated with varying concentrations of these compounds for 24 h. The CCK-8 assay results, presented in Figure 1a, indicated a concentration-dependent increase in the viability of RAW264.7 cells. Notably, compared to the SPS purchased from Sigma-Aldrich, the SOP and SOP-PEG extracted in our laboratory demonstrated a more significant enhancement in macrophage activity. In particular, within the concentration range of 5 µM to 60 µM, both SOP and SOP-PEG exhibited statistically significant increases in cell activity, with SOP-PEG showing the most pronounced effect. In contrast, SPS at a concentration of 60 µM did not show any significant impact, indicating that SOP and SOP-PEG were more effective than SPS in enhancing macrophage activity.

### 3.2. Protamine Enhanced the Secretion of Inflammatory Cytokines in Macrophages

Macrophage viability is generally regarded as an indicator of immune activation, which subsequently drives the production of cytokines such as IL-1β, IL-6, and TNF-α [41,42,43]. These cytokines function as critical intracellular messengers in immune regulation. To assess the immunomodulatory potential of SPS, SOP, and SOP-PEG, their effects on the secretion of inflammatory cytokines by macrophages were investigated. As shown in Figure 1b–e, the addition of protamine at tested concentrations of 10, 20, and 40 μM significantly increased the secretion levels of IL-1β (SPS 40 µM: 32.15 pg/mL; SOP 40 µM: 52.43 pg/mL; SOP-PEG 40 µM: 61.57 pg/mL), IL-6 (SPS 40 µM: 13.22 pg/mL; SOP 40 µM: 21.64 pg/mL; SOP-PEG 40 µM: 29.69 pg/mL), and TNF-α (SPS 40 µM: 109.53 pg/mL; SOP 40 µM: 143.29 pg/mL; SOP-PEG 40 µM: 182.67 pg/mL) in a dose-dependent manner compared to the basal levels observed in the control group. Notably, SOP-PEG exhibited the most significant effect, showing a stronger enhancement compared to SPS and SOP, indicating its greater potential in boosting macrophage immune activity.

### 3.3. Protamine Enhanced NO Secretion Level and Phagocytosis Activity in Macrophages

Nitric oxide (NO) production and phagocytic activity are hallmark indicators of enhanced macrophage activation [44,45]. As shown in Figure 1e, treatment with SPS (40 µM: 10.28 μmol/mL), SOP (40 µM: 14.37 μmol/mL), and SOP-PEG (40 µM: 17.26 μmol/mL) led to increased NO levels in macrophages, with SOP-PEG inducing a significantly greater effect compared to the other groups. To evaluate the impact of protamine on macrophage pinocytosis activity, the neutral red uptake was employed. The results demonstrated that protamine significantly enhanced phagocytic activity in RAW264.7 cells compared to the control group. These findings suggest that protamine possesses the potential to boost the immune activity of RAW264.7 cells, with SOP and SOP-PEG exhibiting superior effects compared to SPS.

### 3.4. Protamine Induced the Generation of ROS in Macrophages

**Figure 2:**
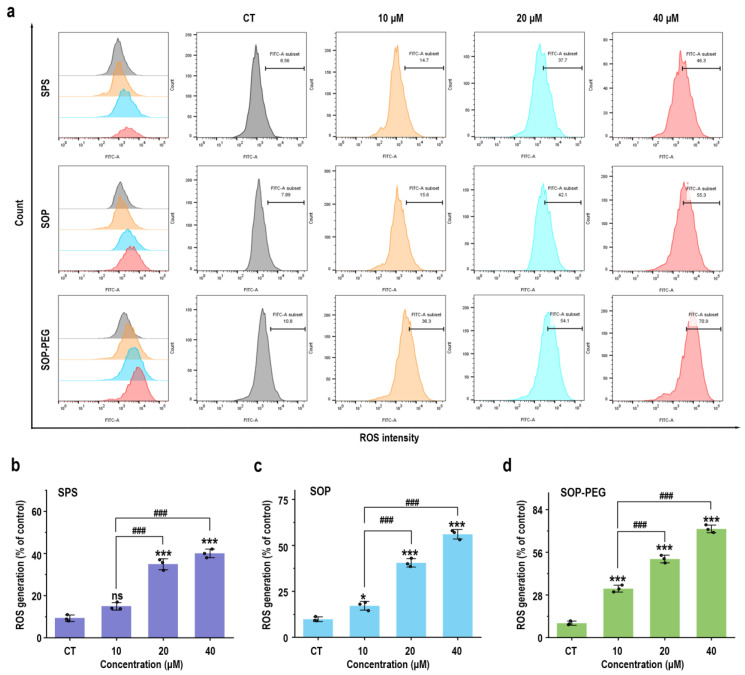
SPS, SOP, and SOP-PEG enhance ROS production in RAW264.7 cells (**a**). Statistical analysis of the percentage of ROS generation in the SPS (**b**), SOP (**c**), and SOP-PEG (**d**) treatment groups. *, and *** represent *p* < 0.05 and *p* < 0.001 compared to the control group, respectively. and ^###^ represent *p* < 0.001 for significant differences between two groups. ns indicates no significant difference.

Reactive oxygen species (ROS) play a crucial role in the innate immune response as key signaling molecules that regulate the secretion of inflammatory factors and modulate the intensity of the immune response [46]. To determine whether protamine induced ROS production in macrophages, RAW264.7 cells treated with protamine were labeled with fluorescent probes, and a flow cytometry (FCM) analysis was performed. The results revealed that protamine (SPS 40 µM: 44.4; SOP 40 µM: 55.3; SOP-PEG 40 µM: 70.9) significantly enhanced ROS production in macrophages (Figure 2). Furthermore, SOP and SOP-PEG treatments resulted in a pronounced accumulation of ROS compared to the control group. Similarly, SPS treatment also induced ROS production in macrophages, albeit to a lesser extent. Notably, SOP-PEG exhibited the most significant effect in ROS-induced immune activity enhancement, showing a stronger impact than SPS and SOP. This further confirms the critical role of SOP-PEG in regulating macrophage immune responses.

### 3.5. ROS Plays a Crucial Role in Promoting Macrophage Immune Activity by Protamine

**Figure 3:**
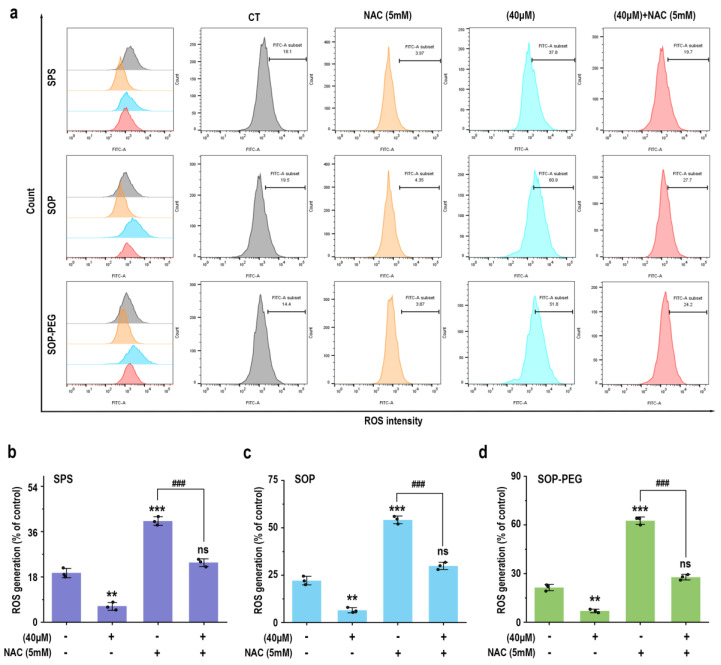
Effects of NAC (5 mM) pretreatment on ROS production in RAW264.7 cells. (**a**) Representative ROS production in RAW264.7 cells with or without NAC pretreatment. (**b**–**d**) Statistical analysis of ROS production percentages in cells pretreated with or without NAC. **, and *** represent *p* < 0.01 and *p* < 0.001 compared to the control group, respectively. ^###^ represent *p* < 0.001 for significant differences between two groups. ns indicates no significant difference.

**Figure 4:**
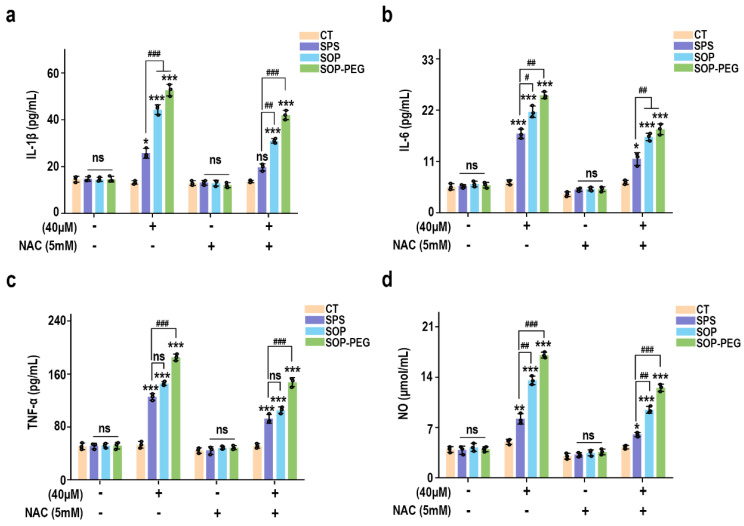
Effects of NAC (5 mM) pretreatment on the secretion of IL-1β (**a**), IL-6 (**b**), TNF-α (**c**), and NO (**d**) in RAW264.7 cells. *, **, and *** represent *p* < 0.05, *p* < 0.01, and *p* < 0.001 compared to the control group, respectively. ^#^, ^##^, and ^###^ represent *p* < 0.05, *p* < 0.01, and *p* < 0.001 for significant differences between two groups. ns indicates no significant difference.

To further elucidate the role of ROS in protamine-induced macrophage immune activity, macrophages were pre-incubated with the ROS scavenger NAC to evaluate whether it could attenuate protamine’s effects. As shown in Figure 3, NAC significantly reduced ROS accumulation in the protamine-treated group. Moreover, compared to the protamine-alone treatment group, the combined treatment of NAC and protamine markedly diminished the production of IL-1β (SPS 40 µM: 19.27 pg/mL; SOP 40 µM: 30.73 pg/mL; SOP-PEG 40 µM: 41.28 pg/mL) (Figure 4a), IL-6 (SPS 40 µM: 11.38 pg/mL; SOP 40 µM: 16.24 pg/mL; SOP-PEG 40 µM: 18.97 pg/mL) (Figure 4b), TNF-α (SPS 40 µM: 86.64 pg/mL; SOP 40 µM: 105.16 pg/mL; SOP-PEG 40 µM: 143.57 pg/mL) (Figure 4c), and NO (Figure 4d). These results demonstrate that ROS regulates macrophage activity through oxidative stress responses, promoting the secretion of inflammatory cytokines and enhancing immune responses. This confirms that ROS is a critical mediator of protamine’s regulatory effects on macrophage immune activity. The precise levels and distribution of ROS are essential for modulating macrophage immune functions, enabling effective immune defense while preventing potential autologous damage.

### 3.6. Protamine Induces the Antioxidant Activity of Macrophages

**Figure 5:**
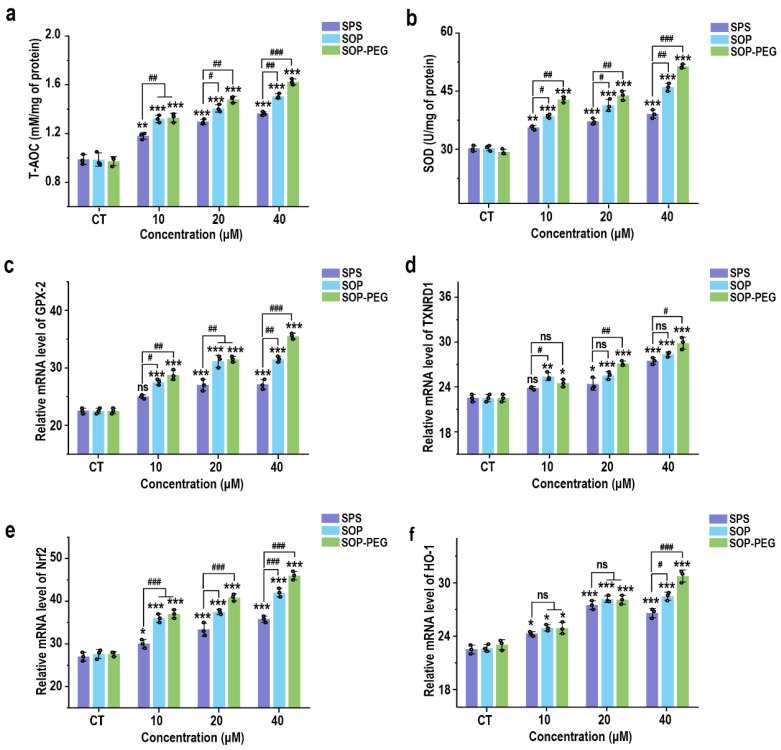
Effects of SPS, SOP, and SOP-PEG on antioxidant capacity (**a**), SOD activity (**b**), and mRNA expression of antioxidant-related genes (GPX-2 (**c**), TXNRD1 (**d**), Nrf2 (**e**), and HO-1 (**f**)) in RAW264.7 cells. *, **, and *** represent *p* < 0.05, *p* < 0.01, and *p* < 0.001 compared to the control, respectively. ^#^, ^##^, and ^###^ represent *p* < 0.05, *p* < 0.01, and *p* < 0.001, indicating significant differences between the two groups. ns indicates no significant difference.

Although ROS activation is essential for immune response, it also regulates excessive inflammation through a negative feedback mechanism [47,48]. Previous studies have demonstrated that ROS can induce the expression of antioxidant systems, thereby reducing ROS levels and limiting the intensity of the immune response to prevent tissue damage [49,50]. As expected, SPS, SOP, and SOP-PEG all enhanced the antioxidant capacity of macrophages (Figure 5a) and increased SOD activity (Figure 5b). Additionally, at the mRNA level, protamine promoted the expression of antioxidant-related genes, including GPX-2 (Figure 5c), TXNRD1 (Figure 5d), Nrf2 (Figure 5e), and HO-1 (Figure 5f).

### 3.7. Effects of Protamine on PI3K/AKT Signaling Proteins

**Figure 6:**
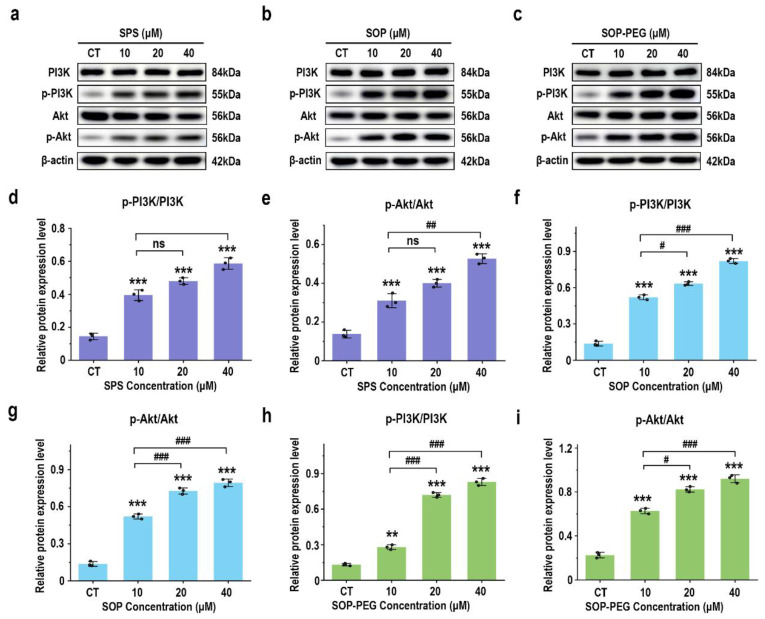
Effects of SPS (**a**), SOP (**b**), and SOP-PEG (**c**) on the expression of proteins related to the PI3K-Akt signaling pathway by Western blot. Statistical analysis of PI3K, p-PI3K, Akt, and p-Akt protein expression (**d**–**i**) was performed. **, and *** represent *p* < 0.01, and *p* < 0.001 compared to the control, respectively. ^#^, ^##^, and ^###^ represent *p* < 0.05, *p* < 0.01, and *p* < 0.001, indicating significant differences between the two groups. ns indicates no significant difference.

The PI3K-Akt signaling pathway is key to modulating macrophage immune function, such as enhancing inflammatory factor secretion, ensuring cell survival, regulating phagocytosis, and supporting antioxidant defenses [51,52]. The Western blot analysis revealed that protamine treatment (10, 20, 40 μM) significantly increased the expression levels of the growth-related proteins PI3K and Akt in macrophages compared to the control group, in a dose-dependent manner (Figure 6). Notably, the SOP-PEG treatment showed a more pronounced effect in enhancing p-PI3K (SPS 40 µM: 0.58; SOP 40 µM: 0.79; SOP-PEG 40 µM: 0.85) and p-Akt (SPS 40 µM: 0.52; SOP 40 µM: 0.81; SOP-PEG 40 µM: 0.96) expression compared to SPS and SOP.

### 3.8. Effect of Protamine on Anti-Apoptosis Signaling Proteins

**Figure 7:**
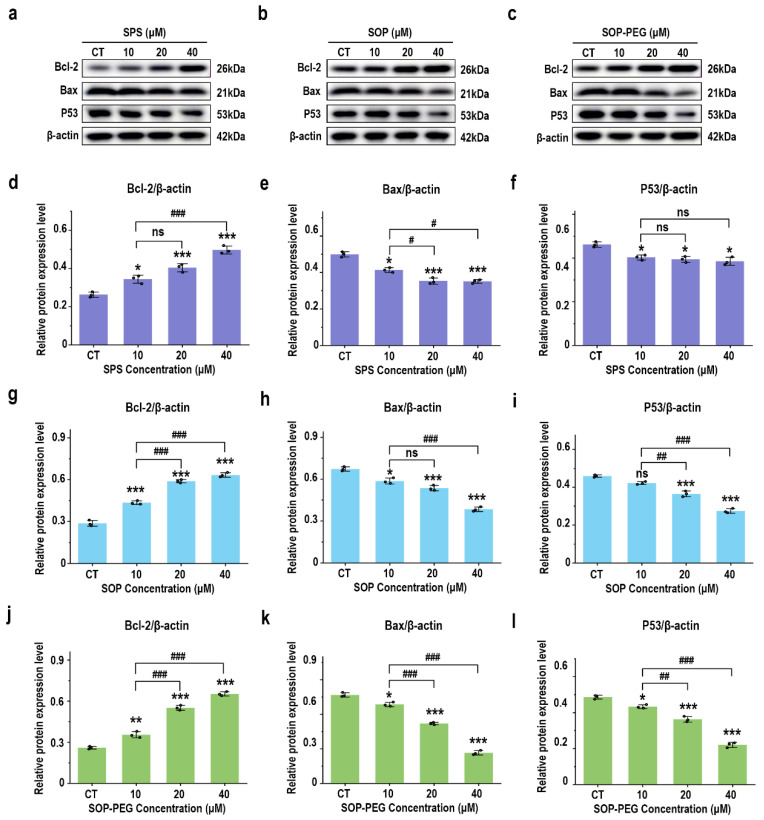
Effects of SPS (**a**), SOP (**b**), and SOP-PEG (**c**) on the expression of proteins involved in anti-apoptosis signaling by Western blot. Statistical analysis of Bcl-2, Bax, and P53 protein expression levels (**d**–**l**) is shown. *, **, and *** represent *p* < 0.05, *p* < 0.01, and *p* < 0.001 compared to the control, respectively. ^#^, ^##^, and ^###^ represent *p* < 0.05, *p* < 0.01, and *p* < 0.001, indicating significant differences between the two groups. ns indicates no significant difference.

The PI3K-Akt pathway activates anti-apoptotic factors and inhibits pro-apoptotic proteins, thereby prolonging macrophage survival and enhancing their role in the immune response [53,54]. As anticipated, treatment with SPS, SOP, and SOP-PEG significantly increased the protein expression of the anti-apoptotic gene Bcl-2(SPS 40 µM: 0.51; SOP 40 µM: 0.61; SOP-PEG 40 µM: 0.67) in macrophages (Figure 7a–d,g,j), while markedly inhibiting the expression of the pro-apoptotic gene Bax (SPS 40 µM: 0.54; SOP 40 µM: 0.36; SOP-PEG 40 µM: 0.27) (Figure 7a–c,e,h,k). The P53 protein plays a multifaceted role in macrophage immune activity, participating in inflammation, apoptosis, cell cycle regulation, and antioxidant responses [55]. The Western blot analysis revealed that SOP (40 µM: 0.55) (Figure 7b,i) and SOP-PEG (40 µM: 0.41) (Figure 7c,l) significantly inhibited the expression of P53 in macrophages. Interestingly, compared to the control group, the changes in P53 protein expression in the SPS (40 µM: 0.75) treatment group were not concentration-dependent (Figure 7a,f).

### 3.9. Effect of Protamine on Cell Cycle in Macrophages

**Figure 8:**
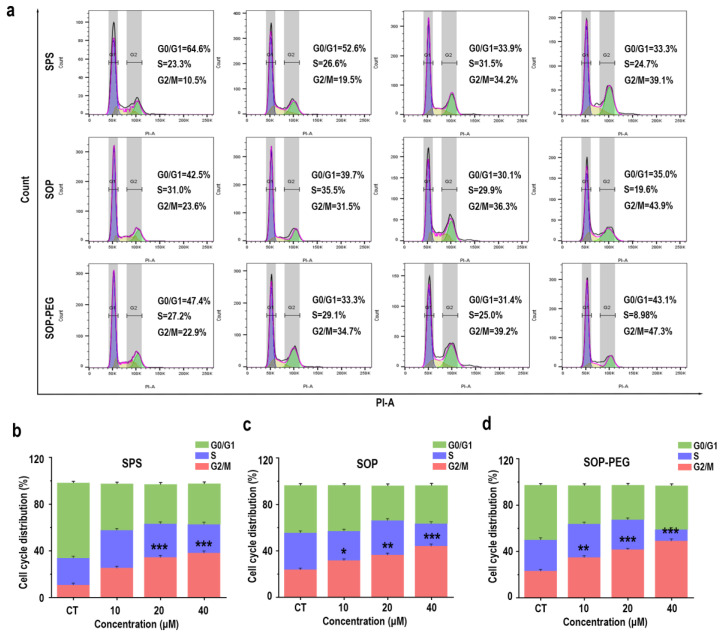
Effects of SPS, SOP, and SOP-PEG on cell cycle progression in RAW264.7 cells (**a**). Statistical analysis of the percentage of cells in different cell cycle phases following treatment with SPS (**b**), SOP (**c**), and SOP-PEG (**d**). *, **, and *** represent *p* < 0.05, *p* < 0.01, and *p* < 0.001 compared to the control, respectively. ns indicates no significant difference.

In addition to the PI3K-Akt pathway and its anti-apoptotic effects, the connection between the cell cycle and macrophage immune activity is also crucial [56,57]. The PI3K/Akt pathway not only promotes cell survival but also facilitates normal cell cycle progression by inhibiting apoptosis-related proteins [58]. Protamine has been shown to enhance macrophage immune activity [59], and it may exert this effect by regulating the cell cycle. The flow cytometry analysis revealed a notable increase in the proportion of cells in the G2/M (SPS 40 µM: 39.1%; SOP 40 µM: 43.9%; SOP-PEG 40 µM: 47.3%) phase following protamine treatment (Figure 8). This indicates that protamine promotes cell cycle progression, specifically facilitating the transition into the G2/M phase.

### 3.10. Effect of Protamine on G2/M Phase-Related Proteins in Macrophages

**Figure 9:**
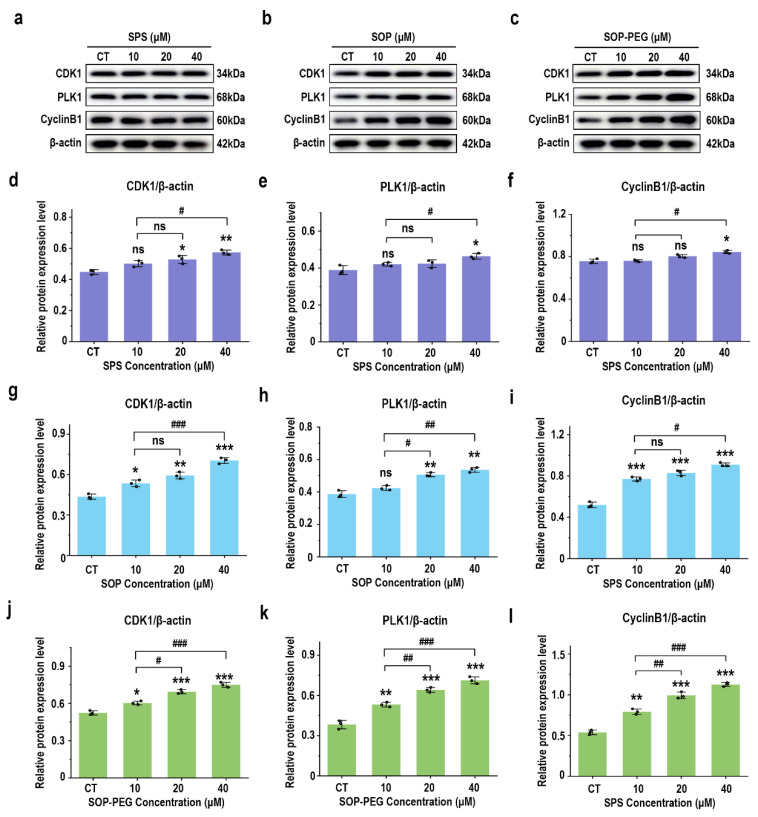
Effects of SPS (**a**), SOP (**b**), and SOP-PEG (**c**) on the expression of key proteins involved in the G2/M phase transition. Statistical analysis of CDK1, cyclin B1, and PLK1 protein expression (**d**–**l**) was conducted. *, **, and *** represent *p* < 0.05, *p* < 0.01, and *p* < 0.001 compared to the control, respectively. ^#^, ^##^, and ^###^ represent *p* < 0.05, *p* < 0.01, and *p* < 0.001, indicating significant differences between the two groups. ns indicates no significant difference.

A further WB analysis was performed to examine the expression of key cyclins involved in the G2/M phase transition. As shown in Figure 9, compared with the SPS treatment group, the protein levels of CDK1 (SPS 40 µM: 0.57; SOP 40 µM: 0.68; SOP-PEG 40 µM: 0.77), cyclin B1 (SPS 40 µM: 0.83; SOP 40 µM: 0.92; SOP-PEG 40 µM: 1.13), and PLK1 (SPS 40 µM: 0.45; SOP 40 µM: 0.55; SOP-PEG 40 µM: 0.74) were significantly increased following treatment with SOP and SOP-PEG. These cyclins are crucial for controlling the transition from the G2 to M phase [60,61,62]. From these findings, we infer that protamine may enhance macrophage proliferation and immune activity by modulating the expression of these cyclins, particularly during the G2/M phase transition.

## 4. Discussion

Protamine, a basic polypeptide, has attracted considerable attention in recent years for its role in enhancing immune activity [63,64]. Initially utilized for anticoagulation and DNA stabilization [65], protamine’s application in modulating immune activity has become increasingly recognized. In parallel, PEGylation—a modification that involves conjugating polyethylene glycol (PEG) to proteins—has proven to significantly enhance the stability, efficacy, and safety of therapeutic proteins, making it a preferred strategy in the development of protein-based drugs [30,66]. In this study, we observed that protamine and its derivatives (SPS, SOP, and SOP-PEG) enhanced the activity of RAW264.7 macrophages and stimulated the release of various pro-inflammatory cytokines such as IL-1β, IL-6, and TNF-α (Figure 1). Among these, SOP and SOP-PEG demonstrated greater efficacy compared to SPS. These cytokines play critical roles in immune signaling and activation, further recruiting other immune cells to amplify the immune response. This indicates that protamine derivatives, particularly PEGylated forms, could serve as promising immunomodulatory agents with significant potential for therapeutic applications.

In macrophages, the generation of ROS serves as a crucial defense mechanism that aids in the clearance of pathogens and damaged cells [67,68]. It has become a major focus in the study of tumor immunology, inflammatory responses, and pathogen defense. The primary sources of ROS in macrophages are mitochondrial oxidative phosphorylation and the NADPH oxidase (NOX) system [69]. Other sources include lipoxygenases (LOX), cyclooxygenases (COX), and peroxidases [70]. ROS can directly oxidize proteins, lipids, and nucleic acids within pathogens, exerting a cytotoxic effect. Moreover, ROS influences the secretion of inflammatory cytokines by modulating signaling pathways such as PI3K-Akt, NF-κB, and Nrf2 [71].

Protamine has been demonstrated to stimulate ROS generation in RAW264.7 macrophages (Figure 2). Furthermore, pre-incubation with the ROS scavenger NAC confirmed that ROS played a key role in the ability of protamine to enhance macrophage immune activity (Figure 3 and Figure 4). However, an overproduction of ROS can harm cell membranes, proteins, and DNA, potentially resulting in cell death [72]. Therefore, cells require effective antioxidant mechanisms to maintain a balance in ROS levels and prevent excessive cellular damage [73]. Our results indicate that protamine can activate the antioxidant system in macrophages (Figure 5), increasing the expression of antioxidant genes like HO-1 and NQO1, thereby protecting macrophages from oxidative damage. This suggests that protamine not only stimulates ROS production but also promotes cellular defense mechanisms, providing a balance between immune activation and protection from oxidative stress.

Macrophage immune activity is governed by an intricate network of signaling pathways, which integrate signals from cytokines, pathogen-associated molecular patterns (PAMPs), damage-associated molecular patterns (DAMPs), metabolic conditions, and other influences [74,75]. Signal transduction plays a critical role in the regulation of macrophage immune activity. Macrophages sense external stimuli through a series of signaling pathways, which regulate their differentiation, function, and interactions with other immune cells [4]. This enables macrophages to perform diverse roles in host defense, tissue repair, inflammatory responses, and tumor immunity. For example, Fang et al. found that arsenic trioxide (ATO) could enhance macrophage anti-apoptotic capacity through the Akt-mediated mTOR signaling pathway, thereby increasing macrophage survival and improving its immunoregulatory activity [76].

The PI3K/Akt pathway plays a central role in the regulation of macrophage immune activity [58]. This pathway responds to external stimuli (such as cytokines and pathogens) and controls cellular metabolism, the expression of inflammatory factors, and polarization states, coordinating a variety of macrophage functions [4,53]. Our findings demonstrate that protamine and its derivatives effectively activate the PI3K/Akt signaling pathway in macrophages (Figure 6). The marked increase in Akt phosphorylation levels indicates that this signaling pathway serves a pivotal role in the immune regulatory process mediated by protamine in macrophages. This activation contributes to enhanced cellular responses, such as increased expression of inflammatory cytokines and altered metabolic states, ultimately coordinating a more robust immune response. Notably, among all treatment groups, SOP-PEG induced the highest level of Akt phosphorylation, indicating its most significant effect on activating the PI3K/Akt signaling pathway. This further confirms the advantage of SOP-PEG in enhancing macrophage immune activity, potentially amplifying the immune response by more effectively regulating cellular signaling pathways, making it a promising immunostimulant.

Protamine and its derivatives significantly enhance the survival capacity of macrophages under high-stress conditions, which is closely linked to the activation of anti-apoptotic signaling pathways. Our results (Figure 7) demonstrate that protamine upregulates the expression of the anti-apoptotic protein Bcl-2, while markedly reducing the activity of Bax, thereby inhibiting macrophage apoptosis. Notably, among all treatment groups, SOP-PEG exhibited the most pronounced regulatory effect, characterized by the highest Bcl-2 expression and the lowest Bax activity. This further suggests that SOP-PEG holds greater potential in enhancing macrophage survival and function, possibly by more effectively inhibiting apoptotic signaling pathways to amplify the immune response. Anti-apoptotic signaling pathways exhibit complex interactions with other signaling pathways, which are fundamental for maintaining cellular homeostasis, regulating stress responses, and determining cellular fate (such as survival, differentiation, or death) [77,78]. In the PI3K/Akt pathway, Akt promotes p53 degradation by regulating the activity of MDM2, thereby inhibiting p53-mediated apoptosis [54]. Akt also maintains mitochondrial membrane potential stability by upregulating Bcl-2 and inhibiting pro-apoptotic proteins such as Bad and Bax, thus antagonizing apoptotic signals [53,79].

Furthermore, this study demonstrates that spermine and its derivatives significantly influence the macrophage cell cycle primarily by enhancing cell proliferation (Figure 8). The activation of the PI3K/Akt pathway results in elevated expression of cell cycle-associated proteins, including CDK1, PLK1, and cyclin B1 (Figure 9), thereby positively regulating the cell cycle. This suggests that spermine accelerates the G2/M transition, reduces cell cycle arrest, and allows macrophages to complete mitosis more rapidly, thereby enhancing their proliferation efficiency. The interaction between the PI3K-Akt pathway, anti-apoptotic signals, and the cell cycle is essential, as they collaborate to enhance cell survival and adaptability. Under stress conditions, PI3K-Akt signaling upregulates anti-apoptotic molecules such as Bcl-2 to protect cell survival, while ensuring the expression of cell cycle-related genes remains unaffected, thus maintaining the proper progression of the cell cycle [80,81]. This mechanism may represent a key pathway by which spermine promotes macrophage proliferation, laying the foundation for improved immune function.

Overall, the close interconnection between the PI3K-Akt signaling pathway, anti-apoptotic signals, and the cell cycle provides a foundation for cell survival, proliferation, and functional maintenance. Although this study emphasizes the key role of protamine (SPS, SOP, and SOP-PEG) in regulating macrophage immune function, it has limitations, mainly confined to in vitro models, lacking in vivo validation and exploration of potential targets. Future research will further validate the immune regulatory mechanisms of protamine in vivo, with a focus on analyzing immune signaling pathways such as PI3K-Akt, MAPK, and NF-κB. Additionally, the study will explore the binding characteristics of protamine with macrophage surface receptors (such as TLRs and CD14) to identify specific targets and optimize derivative design.

## 5. Conclusions

**Figure 10:**
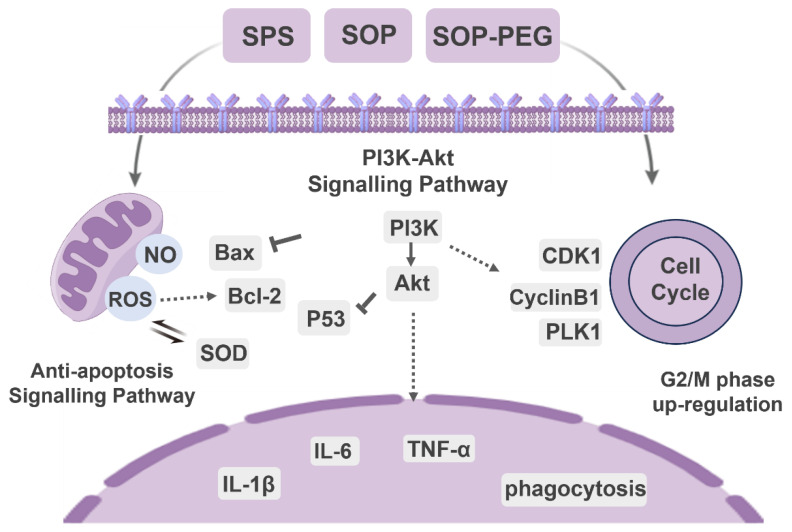
The molecular signaling mechanism of protamine (SPS, SOP and SOP-PEG) regulating immune function in macrophages.

In conclusion, our study demonstrated that arginine-rich alkaline proteins (SPS, SOP, and SOP-PEG) regulate macrophage immune function by modulating immune activity through PI3K/Akt signaling pathway activation (Figure 10). These proteins enhance the secretion of inflammatory cytokines and anti-apoptotic capabilities, thereby influencing the cell cycle in RAW264.7 macrophages. Notably, ROS plays a crucial role in boosting the immune activity of macrophages induced by protamine. Moreover, the antioxidant properties of these proteins contribute to immune regulation by maintaining redox homeostasis, preventing excessive oxidative stress-induced damage, and sustaining macrophage viability and function. The regulatory mechanisms of the PI3K/Akt signaling pathway, anti-apoptotic signaling, cell cycle, and oxidative stress response are interconnected, forming a complex network that collectively supports macrophage survival, function, and proliferation, enabling effective immune responses. Notably, SOP-PEG exhibited the most robust effect in regulating RAW264.7 immune activity compared to SPS, potentially due to the enhanced stability and prolonged bioactivity conferred by PEGylation of SOP. Collectively, this study provides a molecular mechanistic basis for the development of novel squid protamine (SOP) immunomodulators, paving the way for new immunotherapy approaches in cancer treatment and inflammatory diseases.

## Figures and Tables

**Table 1:** Primer sequences in RT-PCR used in the measurement of mRNA expression.

**Gene**	**Accession Number**	**Primer Sequence**
GPX-2	NM_030677.2	Forward: 5′-TGGCGTCACTCTGAGGAACAAC-3′
		Reverse: 5′-GCAAGGGAAGCCGAGAACTACC-3′
TXNRD1	NM_001042513.1	Forward: 5′-CTACGCCATCGGTGACATCCTG-3′
		Reverse: 5′-ACAGCCTCTGAGCCAGCAATC-3′
Nrf2	NM_010902.4	Forward: 5′-GTTGCCACCGCCAGGACTAC-3′
		Reverse: 5′-AAACTTGTACCGCCTCGTCTGG-3′
HO-1	NM_010442.2	Forward: 5′-AAGACCGCCTTCCTGCTCAAC-3′
		Reverse: 5′-TCTGACGAAGTGACGCCATCTG-3′
GAPDH	NM_008084.2	Forward: 5′-GGTTGTCTCCTGCGACTTCA-3′
		Reverse: 5′-TGGTCCAGGGTTTCTTACTCC-3′

## Data Availability

Data will be made available on request.
